# Developing a Cold-Related Mortality Database in Bangladesh

**DOI:** 10.3390/ijerph191912175

**Published:** 2022-09-26

**Authors:** Md. Mahbub Alam, A.S.M. Mahtab, M. Razu Ahmed, Quazi K. Hassan

**Affiliations:** 1 Department of Physics, Khulna University of Engineering and Technology, Khulna 9203, Bangladesh; 2 Schulich School of Engineering, University of Calgary, Calgary, AB T2N 1N4, Canada

**Keywords:** campfire, climate (temperature), cold wave, demography, disaster, poverty, public health, socioeconomic

## Abstract

The aim of this study was to develop a database of historical cold-related mortality in Bangladesh using information obtained from online national newspapers and to analyze such data to understand the spatiotemporal distribution, demographic dynamics, and causes of deaths related to cold temperatures in winter. We prepared a comprehensive database containing information relating to the winter months (December to February) of 2009–2021 for the eight administrative divisions of Bangladesh and systematically removed redundant records. We found that 1249 people died in Bangladesh during this period due to cold and cold-related illnesses, with an average of 104.1 deaths per year. The maximum number of cold-related deaths (36.51%) occurred in the Rangpur Division. The numbers were much higher here than in the other divisions because Rangpur has the lowest average monthly air temperature during the winter months and the poorest socioeconomic conditions. The primary peak of cold-related mortality occurred during 21–31 December, when cold fronts from the Himalayas entered Bangladesh through the Rangpur Division in the north. A secondary peak occurred on 11–20 January each year. Our results also showed that most of the cold-related mortality cases occurred when the daily maximum temperature was lower than 21 °C. Demographically, the highest number of deaths was observed in children aged six years and under (50.68%), followed by senior citizens 65 years and above (20.42%). Fewer females died than males, but campfire burns were the primary cause of female deaths. Most mortality in Bangladesh was due to the cold (75.5%), cold-triggered illness (10.65%), and campfire burns (5.8%). The results of this research will assist policymakers in understanding the importance of taking necessary actions that protect vulnerable public health from cold-related hazards in Bangladesh.

## 1. Introduction

Cold waves (also known as cold snaps or cold spells) are natural hazards and disasters that cause mortality worldwide [1], including in developing countries such as Bangladesh. A cold wave is a weather phenomenon that cools the air and could cause a rapid decrease in temperature within one day [2]. In the winter season, cold waves from the Himalayas periodically enter Bangladesh from north and north-western directions [3], which causes a rapid reduction in temperature in northern and north-western regions and creates biting cold weather earlier in these regions than in other parts of the country. The cold becomes slightly milder when the wave moves towards the south and southeast of the country. Low temperatures due to cold waves trigger the outbreak of cold-related illnesses, such as pneumonia, influenza, asthma, respiratory complications, diarrhea, and heart diseases [4,5,6], and they cause health hazards and mortality, especially for infants, seniors, and homeless individuals. In the literature, the term ‘mortality’ denotes a demographic usage for the frequency of death in a population [7,8,9,10], that is, deaths (for a given illness) per unit of population (sick and well) [11].

Cold-related mortality occurs in both developing countries (such as Bangladesh) and developed countries [12,13,14,15,16]. It is likely that low temperatures that deviate from climate normals cause higher cold-related mortality in the cold climate countries of the world, not associated with extremely low temperatures [17]. Note that countries where the mean temperature is less than 10 °C are usually considered as cold climate countries [18]. Gasparrini et al. [17] reported that 7.29% of mortality was related to milder but non-optimum cold temperature events (i.e., deviated from climate normals) during 1985–2012, whereas extreme temperatures were responsible for only 0.86% of total mortality, and such cold events occurred 384 times in both cold and non-cold climate countries like Australia, Brazil, Canada, China, Italy, Japan, South Korea, Spain, Sweden, Taiwan, Thailand, the UK, and the USA.

Although the literature indicates that cold-related mortality varies according to a country’s socioeconomic and climatic conditions, the increased frequency of cold waves has led to an increase in mortality in developed countries [15,16]. The increased numbers of cold waves from 1975 to 2003 and their longer durations in Castile-La Mancha, Spain caused increased mortality [15]. In addition, a 1 °C decrease in temperature during the cold season (October–March) was associated with a 1.35% increase in the daily total of cold-related natural deaths in 15 European cities during 1990–2000 [16]. However, a potential link between the poor socioeconomic conditions of mainland Portugal during the recession of 2009 to 2012 and cold-related mortality was found, i.e., winter mortality rates were 30% higher than non-winter mortality rates during the recession, and such rates were significantly lower in the pre-recession (2005–2008) and post-recession (2013–2016) periods [12]. Demographically, males showed a lower cold-related mortality risk than females in developed countries, where children and senior citizens were the most vulnerable [19,20,21]. 

In tropical and sub-tropical South and Southeast Asian countries, several studies have reported increased winter mortality due to lower than typical temperatures [22,23,24,25,26,27,28]. For example, India experienced an increased number of cold waves between 2010 and 2018 [28], where the number of cold-related deaths and mortality risk were much higher during 2001–2013 [23]. Asif et al. [22] reported seasonal deaths in the Chitral District of Pakistan and found that more deaths occurred in winter than in other seasons, with the maximum number occurring in those aged ≥54 years. Another study found that short-term mortality substantially increased among the elderly population in subtropical Asian cities during cold temperatures and spells [27]. Furthermore, studies have reported different risk levels for men and women in different parts of Asia. In China, the mortality risk varies with age, sex, and geography, and increases significantly with cold spells [24]. Here, females were found to be more vulnerable than males during a longer cold spell duration; however, this is because there are more elderly females in China, and they have a higher life expectancy than males [25]. However, females also have a lower thermoregulatory ability than males due to physiological differences [29]. In contrast to these results, one study showed that males were at a higher risk than females in India [30] because they were more engaged in outdoor activities (such as agriculture, construction, and recreation).

A few studies have focused on cold-related mortality in Bangladesh. For example, a study reported cause-specific mortality in different primary sample units (PSU) of Bangladesh during 2002–2007 using the sample vital registration system (SVRS) established by the Bangladesh Bureau of Statistics (BBS) [31]. They reported that the primary mortality peak occurred from October to February (autumn and winter) and a secondary mortality peak occurred from April to July (summer and monsoon). Other studies found relationships between temperature and mortality in the Matlab Upazila of Chandpur District under the Chattogram Division [32,33], but these studies focused exclusively on one Upazila out of 495 in Bangladesh. Hashizume et al. [32] found a correlation between daily mortality and low temperatures occurring in the preceding week. They also reported a visible increase in all causes of death during low temperatures with a delay of 0–13 days, and each 1 °C decrease in the average daily temperature during the winter months increased the death number by 3.2%. In addition, Babalola et al. [33] correlated infant mortality and low-temperature extremes during 1982–2008 in Matlab Upazila, and the study found that children’s mortality risk was higher during winter months. However, all of these studies in Bangladesh used small areas to determine the seasonality and causes of mortality and linked the results with the magnitude of temperature, but none exclusively investigated the distribution and demographic dynamics of cold-related mortality and illnesses throughout the entire country. The reason for this oversight was likely due to the lack of a complete dataset for the entire country compiled by any organization. Historical data of cold-related deaths in Bangladesh are scattered and only available from differing national and international organizations in limited forms, and no single organisation in Bangladesh provides comprehensive data. Nationally, two non-governmental organisations, i.e., the Foundation for Disaster Forum (DF) [34] and the Network for Information Response and Preparedness Activities on Disaster (NIRAPAD) [35], report cold-related deaths in Bangladesh. In addition, international organisations such as ReliefWeb of the United Nations Office for the Coordination of Humanitarian Affairs (OCHA) [36], the International Federation of Red Cross and Red Crescent Societies (IFRC) [37], and the Emergency Events Database (EM-DAT) of the Centre for Research on the Epidemiology of Disasters (CRED) [38] report cold-related deaths in Bangladesh. However, none of these organisations have reported historically continuous and coherent data on cold-related deaths in Bangladesh. For example, IFRC reported cold-related deaths for the years 2003, 2006, 2007, 2010, 2011, 2013, and 2018; EM-DAT reported cold-related deaths for the years 2003, 2006, 2007, 2010, 2011, 2012, 2018, and 2019; DF reported them for 2010–2018; and NIRAPAD reported them for 2011–2013 and 2015. Moreover, the data reported are not consistent among the organisations. For example, in 2011, IFRC, EM-DAT, DF, and NIRAPAD reported cold-related death data numbers of 50, 62, 133, and 39, respectively.

The Directorate General of Health Services (DGHS) of Bangladesh recently began to provide cold-related death and illness data in the news media for the winter of 2019–2020, based on information collected from hospitals [39]. However, they did not provide cold-related death and illness data for previous years, and there is no guarantee that they will continue to provide such information in the future. In addition, they did not provide the location of occurrences, but they provided a total number for the entire country. Furthermore, they did not report demographic information, such as age and sex, or monthly statistical information. Therefore, there is no existing comprehensive database containing historical cold-related mortality data in Bangladesh. However, major newspapers in Bangladesh publish daily information on cold-related deaths, the causes of death, age, and gender during the winter months, and the local newspaper correspondents collect data from upazila, district, and divisional hospitals. Thus, our overall objective was to comprehend the various aspects related to cold-related mortality in Bangladesh, where mortality includes deaths due to cold-triggered diseases and campfire burns. We considered campfire burns because they are not recreational events during the winter months in Bangladesh; rather people use campfires to keep warm and protect themselves from the bitter cold, especially during cold spells [40]. Finally, the specific objectives of this study were to:

- develop a comprehensive database on division-wise cold-related mortality in Bangladesh during the winter months of 2009–2021 using information from online national newspapers and available reports;
- find a relationship between mortality and the drift of winter temperatures; and
- determine the spatiotemporal variability and demographic dynamics of cold-related mortality in Bangladesh.

## 2. Materials and Methods

### 2.1. Study Area

**Figure 1:**
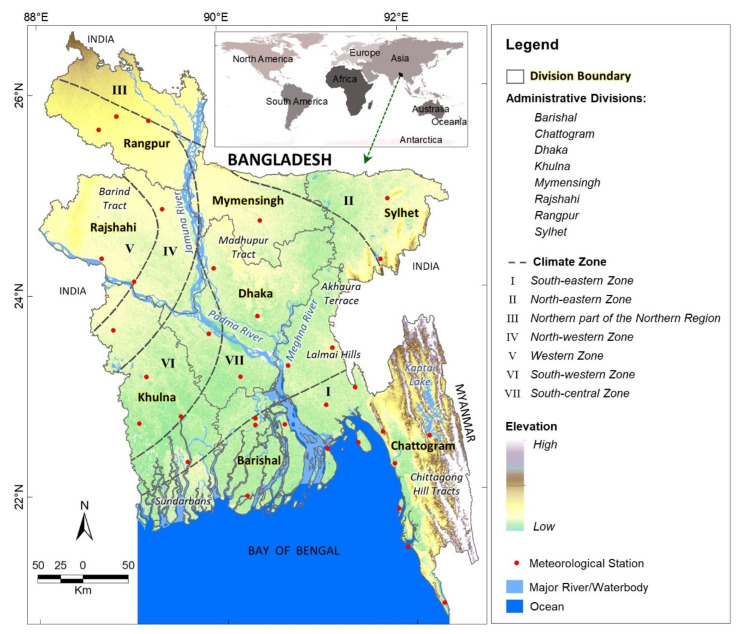
Study area showing the eight administrative divisions and climatic sub-zones of Bangladesh.

Our study area was Bangladesh, which is located in South Asia (see Figure 1) between latitudes 20°34′–26°38′ N and longitudes 88°01′–92°41′ E and which covers an area of 147,540 km^2^ [41]. In 2018, the population of Bangladesh was 164.60 million [42] at a density of 976 person/km^2^ [43]. The country has eight first-order administrative levels (divisions): Barishal, Chattogram, Dhaka, Khulna, Mymensingh, Rajshahi, Rangpur, and Sylhet (see Figure 1). These divisions comprise 64 districts (second-order administrative level), 495 Upazilas (third-order administrative level), and 4562 unions (fourth-order administrative level).

Three distinctive features characterise the physiography of the country: a quaternary delta plain, Pleistocene upland, and tertiary hilly region (in the southeast and northeast). The delta plains of three major rivers (Padma (Ganges), Jamuna (Brahmaputra), and Meghna, and their tributaries) occupy 79% of the country [44,45]. Four upland blocks (Madhupur Tract, Barind Tract, Akhaura Terrace, and Lalmai Hills; see Figure 1) and hilly regions occupy the remaining 9% and 12%, respectively. Topographically, the terrain is nearly flat; the delta plain has elevations of less than 12 m (39 ft) above mean sea level [46], and the hilly region has the highest peak (Bijoy) of 1280 m [41]. The Himalayas lie to the north, and the country is bounded by the Bay of Bengal to the south. The tropical monsoon climate causes rainfall, temperature seasonality, and high humidity throughout the country. The climate can be classified into four seasons: winter (December–February), summer (March–May), monsoon (June–September), and autumn (October–November) [47]. Seven different subzones define the country’s tropical climate, and these climatic subzones are located in the south-eastern, north-eastern, northern part of the northern region, north-western, western, south-western, and south-central areas of the country [48] (see Figure 1). The north-western part of the country experiences extreme temperatures; the daily average temperature is low in winter and high in summer. In winter, wind from the Himalayas (cold waves) enters the country from the north and north-west, and the temperature drops. The annual mean temperature of the country is approximately 25 °C, and the average winter temperature ranges from 17.0 °C to 20.6 °C [47]. The lowest ever recorded minimum temperature was 2.6 °C (recorded on 8 January 2018 in Tetulia Upazila, which is the most extreme north-western Upazila of Bangladesh and under the Panchagarh District of Rangpur Division) [49]. The average annual rainfall varies from 1329 mm in the northwest to 4338 mm in the northeast, and the western part receives much lower amounts of rainfall than the rest of the country [50,51].

### 2.2. Data and Methods

**Figure 2:**
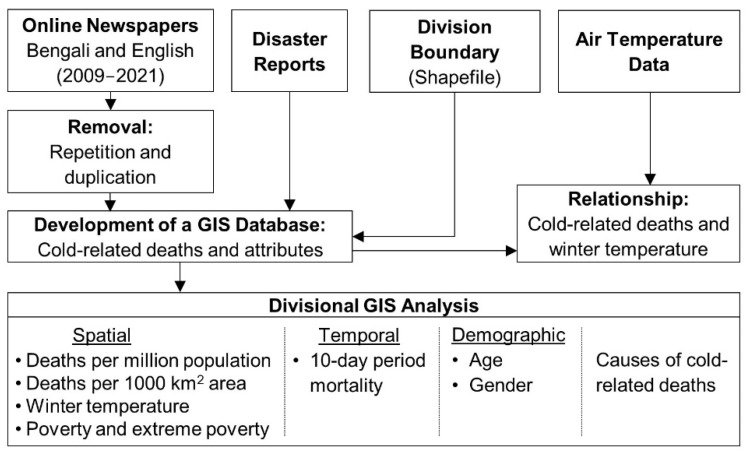
A schematic diagram of the GIS database development and the methodology followed in this study.

A schematic diagram of the geographic information system (GIS) database development and the methods used in this study are shown in Figure 2. As there was no available national-scale database of cold-related deaths during the winter months for the study area, we developed a database by compiling the information collected from the following sources, which involved substantial and labour-intensive work:

- Two daily Bengali national newspapers and three daily English national newspapers. The Bengali newspapers were *Prothom Alo* [52] and *Ittefaq* [53], and the English newspapers were *The Daily Star* [54], *Daily Observer* [55], and the *Dhaka Tribune* [56];
- Disaster reports from NIRAPAD [35] and DF [34].

Online national newspapers from December 2009 to February 2021 were the primary sources of information about cold-related deaths attributed to cold-triggered diseases and campfire burns. In this respect, the development of databases based on newspapers is not a new concept; for example, previous studies used newspapers to collect and compile information about lightning-related deaths [57,58]. For the cold-related mortality data obtained in this study, we physically scanned online versions of *The Daily Star* and *Prothom Alo* for the winter months (December–February) from 2009 to 2021, and the *Dhaka Tribune*, *Daily Observer,* and *Ittefaq* from 2015 to 2021. The online versions of *The Daily Star*, *Prothom Alo,* and the remaining newspapers were available in 2003, 2009, and 2015, respectively. We set our data collection period from December 2009 to February 2021 by considering the availability of reported data from at least two online newspapers for cross-validation. Therefore, although *The Daily Star* became available online in 2003, it was the only online newspaper until 2008, and therefore we did not consider the period 2003–2008 in this study.

Cold-related mortality data were collected for all administrative levels (division, district, and Upazila) using a standardized form to record the location, gender, age, date, and cause of death. However, we identified that data recorded at district and Upazila levels were not conclusive because the locations of deaths were missing; therefore, we compiled the data at the division level. We also noted that newspapers occasionally repeated the numbers of cold-related deaths. To eliminate such repetitions, we cross-checked the location of the deaths (i.e., union-level rural clinics/hospitals, Upazila health complex, district level hospitals, and medical college hospitals) to confirm the individuality of the date, age, gender, and cause. In addition, we found that information about cold-related deaths was only available in newspapers from 1 December to 10 February for each winter season. Therefore, we could not compile data from 11 to 28/29 February during the winter seasons of 2009–2021.

Nevertheless, once we collected the cold-related mortality data for the divisions, we used a GIS to integrate it as attributes to a spatial division shapefile obtained from the World Bank Open Data [59] to develop a GIS database. We used the GIS database to perform spatial, temporal, and demographic analyses with the aim of understanding the spatiotemporal dynamics and potential socioeconomic factors related to cold-related deaths in Bangladesh. For the spatial analysis, we applied a standard deviation method to the cold-related death data to rank the position of each division in the country. We also compared the total number of cold-related deaths with the poverty (and extreme poverty) ratios of the divisions and monthly average daily minimum air temperature (°C) in the winter months (December, January, and February) for the period 2009–2021. To rank cold-related deaths, we normalized the number of deaths per million population and 1000 km^2^ area of each division according to the 2011 census [43]. We performed normalisation on the GIS database and prepared a map using the standard deviation (SD) method. SD is calculated as the square root of variance that determines each data point’s deviation relative to the mean [60]. We also generated poverty and extreme poverty maps to understand the ranking of divisions and the spatial distribution of variability. Here, we acquired poverty and extreme poverty ratios from the DataBank of The World Bank Open Data [59] and integrated them into the GIS database before the analysis. It is of note that the definition of poverty used here refers to anyone who lives below the international poverty line and survives on less than US$ 1.90 per day, and anyone living on US$ 1.25 or less a day is in extreme poverty [43,61]. These data were available at the district level and were aggregated for the divisions. In addition, we collected temperature data (daily minimum) from 34 meteorological stations in the Bangladesh Meteorological Department (BMD) to calculate the average minimum temperature in each division for each winter month (see Figure 1). To understand the temperature distribution, we applied an interpolation technique (i.e., kriging) to generate temperature isolines for the winter months. Next, we prepared line and bar graphs to visualise the relationship between cold-related deaths and winter temperatures. We also performed a qualitative analysis to understand the similarity between our calculated average minimum temperature of each division and the temperature reported in the newspapers.

For the temporal analysis, we sliced the three winter months of every year into approximate 10-day periods (i.e., a total of seven periods from 1 December to 10 February, with three periods each in December and January, and one period in February) to estimate the division-wise cold mortality during 2009–2021. Initially, we organized the mortality data of each temporal slice for the districts, which were aggregated to the division level. These data helped us analyze cold mortality every 10/11 days during the winter months and their inter-annual distribution. To understand the demographic dynamics, we investigated the division-wise distribution of age and sex associated with cold mortality during 2009–2021. This study adopted the age classification scheme of the World Bank (that is, aged six and under, aged seven–14, aged 15–64, and aged 65 and over) [59]. We also included another category (*age not mentioned*) for when age information was missing. In the case of gender, the data were categorized into male, female, children (six years and under), and *gender not mentioned* (for when gender information was not available). In addition, we compiled the data in a tabular form to analyze the causes of death (such as cold disease and campfire burn) and their spatial and temporal distributions in the divisions.

## 3. Results

### 3.1. Spatial Distribution of Cold-Related Mortality and Its Associated Factors

#### 3.1.1. Cold-Related Deaths

**Figure 3:**
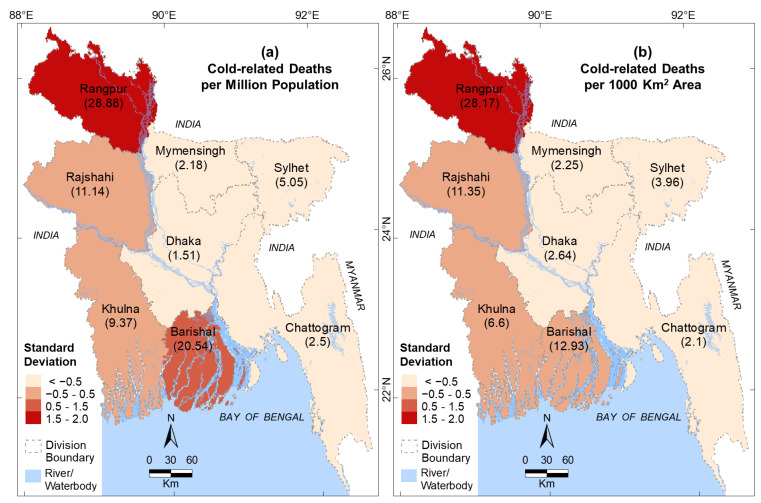
Standard deviations for (**a**) cold-related deaths per million population and (**b**) per 1000 km^2^ area in the divisions of Bangladesh during 2009–2021. The numbers in brackets represent the specific absolute value of the division.

Figure 3a,b show the SD of normalized cold-related deaths per million population and 1000 km^2^ area, respectively, in the eight divisions during the winter seasons of 2009–2021. Rangpur Division had the highest number of cold-related deaths, with a maximum positive SD of 1.5–2.0 in both cases. Barishal Division had the second highest number of cold-related deaths with a positive SD of 0.5–1.5 (Figure 3a) deaths per million population, and it had the mean number of deaths per 1000 km^2^ for the entire country (with a SD of −0.5–0.5) (Figure 3b). The mean numbers of cold-related deaths and deaths per 1000 km^2^ were found in both Rajshahi and Khulna Divisions (Figure 3a,b). However, the Dhaka, Chattogram, Mymensingh, and Sylhet Divisions had lower numbers of deaths (negative SD of <−0.5).

#### 3.1.2. Winter Temperature

**Figure 4:**
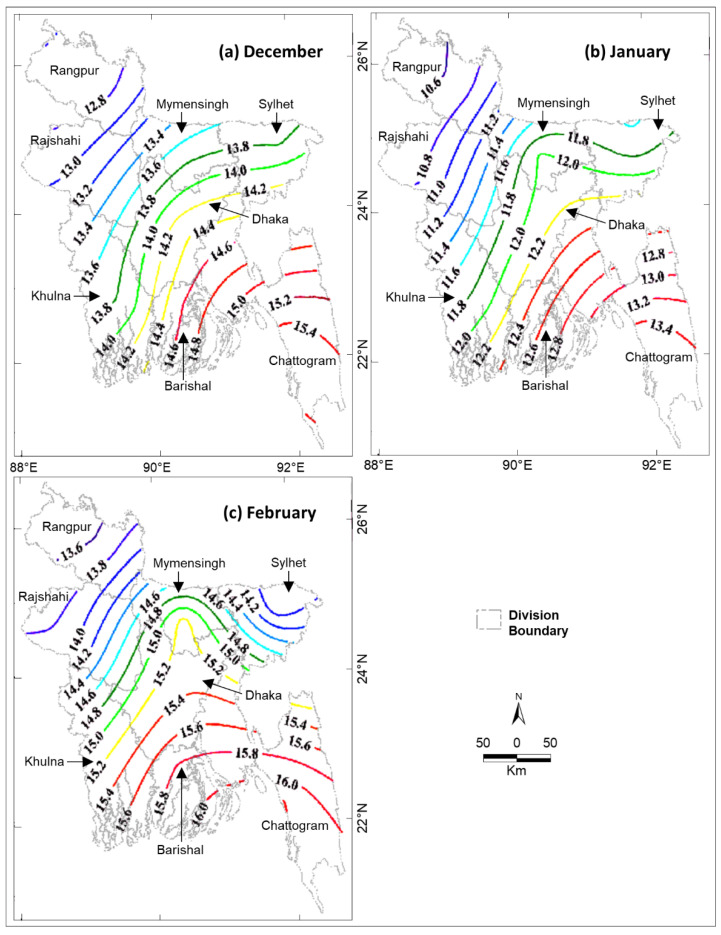
Isolines of the monthly average daily minimum air temperature (°C) in the division during the winter months of 2009 to 2021: (**a**) December, (**b**) January, and (**c**) February.

January was the coldest (Figure 4b) and February was the warmest month (Figure 4c) during winter in Bangladesh from 2009 to 2021. During the winter season, Rangpur Division experienced the lowest monthly average temperatures of 13.70 °C, 10.74 °C, and 12.75 °C in December, January, and February, respectively (see Figure 4a–c). The next coldest division was Rajshahi with temperatures of 13.87 °C, 10.89 °C, and 13.13 °C in December, January, and February, respectively. Chattogram was the warmest division during the winter season with temperatures of 16.25 °C, 13.54 °C, and 15.50 °C in December, January, and February, respectively (see Figure 4a–c).

**Figure 5:**
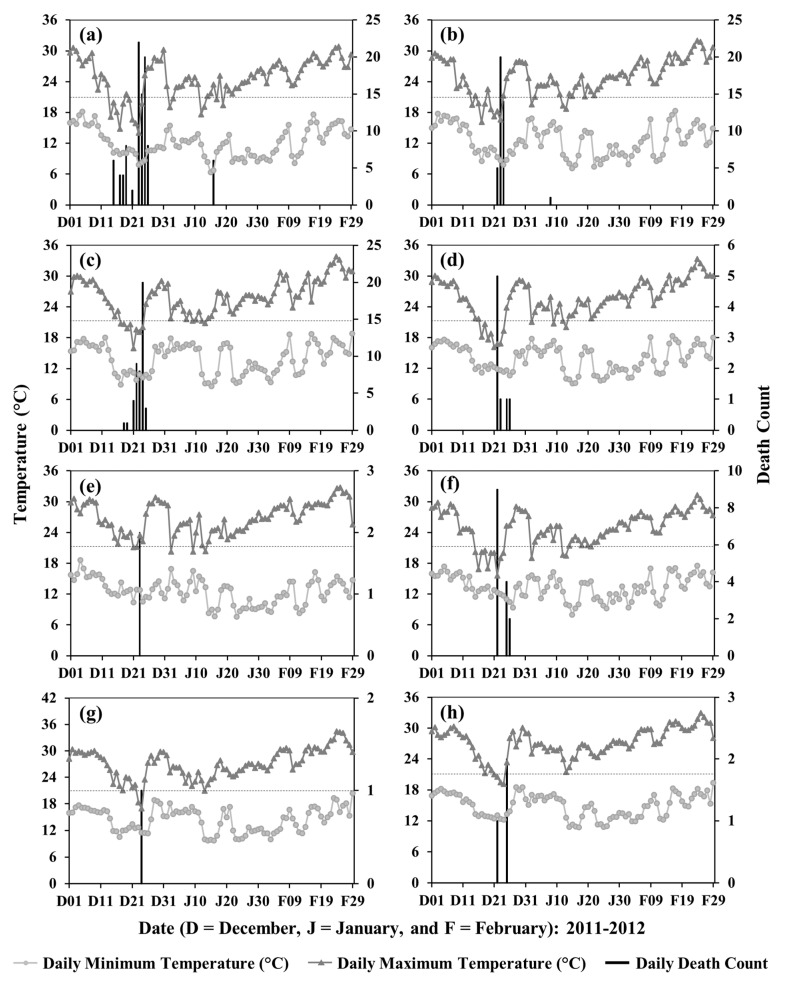
Relationship between cold-related deaths and winter temperatures for 2011–2012 in the divisions of (**a**) Rangpur, (**b**) Rajshahi, (**c**) Khulna, (**d**) Dhaka, (**e**) Sylhet, (**f**) Mymensingh, (**g**) Barishal, and (**h**) Chattogram. Horizontal dotted lines represent moderate cold at 21 °C.

**Figure 6:**
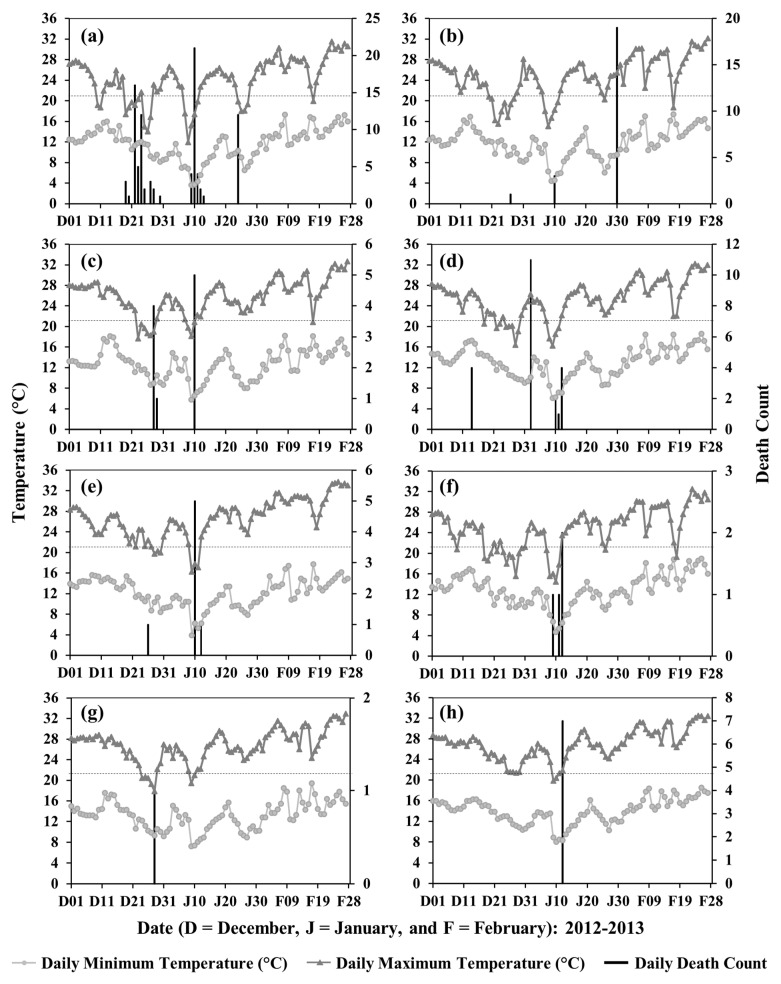
Relationship between cold-related deaths and winter temperatures for 2012–2013 in the divisions of (**a**) Rangpur, (**b**) Rajshahi, (**c**) Khulna, (**d**) Dhaka, (**e**) Sylhet, (**f**) Mymensingh, (**g**) Barishal, and (**h**) Chattogram. Horizontal dotted lines represent moderate cold at 21 °C.

In general, we observed that lower temperatures were associated with cold-related deaths in the divisions of Bangladesh. An exception is the Barishal Division that showed a higher mortality (Figure 3) with the higher temperature in the winter months (Figure 4). In addition, all cold-related deaths in all divisions occurred during or immediately after a period of a few days when the daily maximum temperature was ≤21 °C. Two example periods, 2011–2012 and 2012–2013, are shown in Figure 5 and Figure 6, respectively, when the highest total deaths (i.e., ~31.8%) occurred in the entire study period.

#### 3.1.3. Poverty and Extreme Poverty

**Figure 7:**
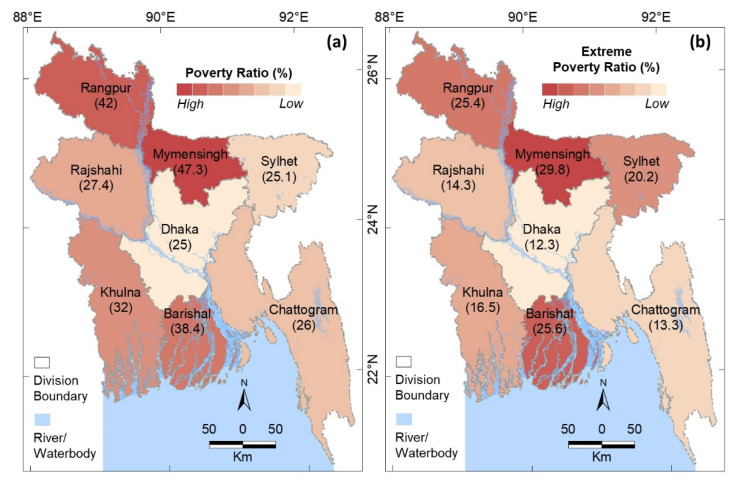
Ratios of the population living in (**a**) poverty and (**b**) extreme poverty in the divisions of Bangladesh. Absolute values are given brackets for each division.

According to the poverty and extreme poverty ratios in Bangladesh, 47.3% and 29.8% of the population in Mymensingh Division live in poverty and extreme poverty, respectively (see Figure 7a,b), which are the highest percentages in Bangladesh. The Rangpur and Barisal Divisions have the second highest poverty levels at 42% and 38.4%, respectively (see Figure 7a), and extreme poverty levels of 25.6% and 25.4%, respectively (see Figure 7b). In contrast, the Dhaka Division has the lowest percentage population living in poverty and extreme poverty.

### 3.2. Temporal Variations in Cold-Related Mortality

We divided the winter months into approximate 10-day intervals and observed two cold-related mortality peaks in Bangladesh, including a primary maximum occurring between 21 and 31 December (509 deaths) and a secondary maximum between 11 and 20 January (287 deaths) (see Table 1). In contrast, the lowest number of deaths were reported at the beginning of the winter season from 1 to 10 December. The highest number of deaths occurred in Rangpur in all periods except from 1 to 10 December, 21 to 31 January, and 1 to 10 February. Of the winter months, the maximum number of deaths occurred in January (669 deaths) and the minimum occurred in February (12 deaths).

More cold-related deaths occurred in the winters of 2009–2010 and 2015–2016, and the lowest number of cold-related deaths occurred in the winters of 2016–2017 and 2020–2021. The highest number of deaths occurred in the winter of 2011–2012 (214 deaths) and the lowest number occurred in the winter of 2016–2017 (18 deaths). There was an average number of 133 cold-related deaths in the winters of 2009–2010 and 2014–2015, and an average number of 75 deaths in the winters of 2015–2016 and 2020–2021. This analysis indicates that cold-related deaths decreased in the most recent years compared with the earlier years of the study period. In a single winter season over the study period, the highest number of total cold-related deaths (117) occurred in Barishal Division during 2018–2019, and the second highest number (95 deaths) occurred in Rangpur in 2011–2012.

### 3.3. Demographic Dynamics of Cold-Related Mortality

The gender-based analysis revealed that the highest cold-related mortality occurred in children (633 deaths) during 2009–2021 (see Table A1), representing approximately 51% of the total deaths in the country. Men and women were in the second and third positions, with 256 and 174 deaths, respectively. The highest number of child deaths (127) occurred in 2018–2019, mostly in the Barishal Division (117). With respect to age groups, the highest number of deaths (633) occurred in children aged six years and under, and the second-highest number (255) occurred in the 65 years and above age group (see Table A2). In contrast, only seven deaths occurred in the seven–14 age group.

### 3.4. Causes of Cold-Related Mortality

Deaths in the winter months of the study period were due to low temperatures and cold-triggered diseases, such as pneumonia, asthma, diarrhea, and campfire-related burns. The highest number of deaths occurred due to low temperatures (943), and of cold-related diseases, pneumonia was the primary cause of death in 61 people (see Table 2). Campfire-related burns caused 73 deaths from 2009 to 2021. Table 3 shows the age and sex of those dying and hospitalized due to campfire-related burns. The highest number of deaths occurred in females within the Rangpur Division (30 deaths) in the 15–64 age group (23 deaths). The number of hospitalisations was also the highest (132) in the Rangpur Division.

## 4. Discussion

### 4.1. Socioeconomic Dimension and Environmental Consequences

Cold-related mortality was the highest in Rangpur Division (36.4%) in the winter months of December, January, and February during the study period of 2009–2021. More than 33% of all deaths occurred in this division, and this percentage was much higher than that of the other divisions (see Figure 3). Several factors could be responsible for the higher number of deaths in the Rangpur Division, including the minimum temperature, population density, and poverty and extreme poverty ratios. The lowest monthly average daily minimum temperatures in the winter months in Rangpur Division (see Figure 4) are due to its spatial proximity to the Himalayas in the north and its greater distance from the ocean in the south (the Bay of Bengal) compared to the other districts. During the winter season, cold fronts (i.e., cold waves) periodically enter Bangladesh from the Himalayas through Rangpur Division in the northwest corridor [3], which causes the proportional decrease in temperatures. The population density of Rangpur Division is not the highest in Bangladesh. Although people are not comparatively the most poor (see Figure 7a) or the most extremely poor (see Figure 7b), this division is at the higher end of the poverty and extreme poverty ratios. Therefore, its spatial location coupled with its high-end poverty status probably caused the higher number of cold-related mortalities in the Rangpur Division compared to the other divisions. The higher poverty could also be responsible for higher mortality in the Barishal Division without lower temperatures (compared to other divisions) during the winter months. 

The nature of cold waves (cold fronts), such as their intensity, duration, and time shift trigger most of the cold-related mortality in Bangladesh. The cold front usually intensifies during January in Bangladesh, and therefore, the highest number of deaths (53.6%) were observed in January during the period 2009–2021. Other studies focusing on neighbouring countries have reported similarly high numbers of deaths in January due to the increasing intensity of cold fronts in the Himalayas. For example, the Tarai region (plain) in Nepal (located between the north of Bangladesh and south of the Himalayas) reported the highest number of national deaths (71.5 %) in January during 1974–2013 [62]. Another study conducted in India documented that 72% of cold-related mortality occurred in January between 1978 and 2014 [63]. In our study, we observed a longer duration (13 days) and time shift of a cold wave occurring in the Rangpur, Rajshahi, and Khulna Divisions during the period from 15 to 27 December 2011 [64], and this could be related to the higher number of cold-related deaths (214) occurring during the winter of 2011–2012 (see Table 1). A similar cold front was observed between 21 and 31 December in the winter of 2018–2019, which caused 117 deaths in Barishal Division (a total of 129 deaths were recorded in Bangladesh as a whole). This was the highest number of deaths observed in Bangladesh in any one year and in any division. With respect to cold-related mortality in Bangladesh, we found that this occurred during moderate cold temperatures when the air temperature was below 21 °C. People can suffer from profound hypothermia when the temperature is below 21 °C (70 °F), which can result in death [65]. Although some studies have found that variable cold temperature thresholds are related to mortality, they certainly have a relationship with the characteristics of different climate zones. Most of the mortality risks reported in the literature are attributable to moderate cold temperatures rather than extreme cold temperatures. Similarly, a study showed thresholds of the mean location-specific temperatures of 24.2, 24.0, and 27.6 °C for Brazil, Taiwan and Thailand, respectively, for cold-related mortality in those tropical and subtropical countries [17].

Demographically, mortality due to winter cold in Bangladesh was found to be higher in two age groups: children aged six years and under and seniors aged 65 years and over (see Table A1 and Table A2). These two age groups also have the highest cold-related mortality in the USA [66], although an Indian study found that more men died than children [63]. Such cold-related mortality in these age groups is due to a lack of natural immunity against various diseases. For example, the highest number of deaths in the December 2018–2019 period in Barisal Division occurred in infants (aged six years and under) in the Sher-E-Bangla Medical College Hospital; they died from cold-related complications [67] associated with diseases such as pneumonia, diarrhoea, and asthma, which occur more often during winter. Pneumonia kills more children worldwide than any other illness [68] and is also the most prominent killer of children in Bangladesh. Worldwide, over two million children under five years of age die of pneumonia annually, which is almost 20% of the total number of deaths occurring in children under the age of five [68]. After young children, the second- and third-highest cold-related mortality rates in Bangladesh occurred in males and females, respectively. The higher rate for men is related to the fact that more men work outdoors than females. Another study also indicated that higher cold-related mortality occurred in males compared to females in regions such as India [63].

Bangladesh is a tropical country with a very short winter, and approximately 61.82% of the population live in rural areas [59]. The weather is hot on most days of the year, and houses are, therefore, well-ventilated to encourage the free flow of air, particularly in rural areas. However, poorer people build houses using low-cost materials, and the insulation is ineffective. Therefore, the temperature within the home is often nearly the same as that outside during the winter months, and people die from being subjected to the continually low inside temperature. Middle income people living in buildings constructed in urban areas suffer because they do not use heaters or air conditioners. However, they are able to afford warmer clothes and blankets to protect themselves from the cold, whereas those living in poverty and extreme poverty in rural areas cannot afford these. Thus, they turn to using campfires to keep warm, as burning materials are readily available in rural areas. This practice causes mortality associated with campfire burns, and we found that greater mortality occurred in the Rangpur Division, which has the lowest winter temperatures in the country. In addition, campfire-related deaths were found to be higher in females. This could be related to the clothes they wear; females in rural areas usually wear a garment traditionally worn in South Asia known as a *Saree (Sari)*, which is an unstitched piece of material that wraps around the body and varies in length between 4.5 m and 9 m and in breadth between 0.6 m and 1.2 m. Women also wear shawls or scarves to warm their upper body during winter. All of these garments and accessories have flammable loose ends that are at high risk of catching fire. Females thus accidentally burn themselves when next to campfires and many subsequently die from severe burns.

We observed that fewer cold-related deaths (see Figure 3) occurred in areas that had low poverty and extreme poverty ratios (see Figure 7), even when lower minimum temperatures occurred in the division, such as in Sylhet (see Figure 4). This indicates that better socioeconomic conditions minimized the number of cold-related mortalities. These results show that as a developing country, Bangladesh needs to focus on increasing economic activities in areas where high numbers of cold-related mortality occur, such as in the Rangpur, Rajshahi, and Barishal Divisions. It is suggested that more agro-based industries could be established in high mortality-prone divisions, where agriculture is the dominant sector. This would improve the socioeconomic conditions of the divisions by minimising the number of people living in poverty and extreme poverty. In addition, Bangladesh should integrate heating systems into new-build houses and infrastructure. Furthermore, developing an operational forecasting system for upcoming cold waves would enable people to prepare for cold-snap events.

### 4.2. Institutional Framework for Mitigating Cold-Related Deaths

Cold-related mortality is high in Bangladesh during the winter months, and appropriate adaptation and mitigation strategies are required to save lives. The country could develop a national-level framework, such as that of the International Centre for Diarrhoeal Disease Research (ICDDRB), which is an example of a system that has nearly eliminated diarrhea in Bangladesh. Research has been conducted under this framework, and an awareness of the dangers and treatment of diarrhea has been developed since the 1960s. The framework was initiated in Dhaka (the capital city of Bangladesh) at the South-East Asia Treaty Organisation (SEATO) Cholera Research Laboratory (CRL), and it was named the ICDDRB in 1978. The country recognises the framework as a national asset that has contributed significantly to health improvements in Bangladesh in recent decades. Research has enabled the development of innovative products and generated evidence that has influenced health policy and practice in Bangladesh and globally. The success of the framework has also created awareness of its impact on public health in Bangladesh. Therefore, to eliminate cold-related mortality in Bangladesh, the government could develop a similar national framework for conducting exclusive research and increasing public awareness. A special care unit dedicated to treating cold-triggered diseases should be established in each district hospital, together with burn-treatment units that have already been established [69]. Both types of treatment facilities should collaborate strongly with the Health Service Department of the Ministry of Health and Family Welfare to enable policy development and implementation, and data and information sharing protocols should be established.

## 5. Conclusions

In this study, we obtained data from online newspapers to develop a GIS database of cold-related mortality in different divisions within Bangladesh during the winters of 2009–2021. The database was then used to determine the spatiotemporal variability, demographic dynamics, and causes of cold-related mortality. We found relationships between cold-related mortality and the factors like daily average lower temperatures during the winter months, the population density, and socioeconomic conditions (such as poverty and extreme poverty). We also observed that most of the deaths occurred when the daily maximum temperature was less than or equal to 21 °C. There was a total of 1249 cold-related death during the study period. The highest mortality occurred in the Rangpur Division, where the daily average temperatures were the lowest during the winter months. Deaths were related to the spatial location and poor socioeconomic conditions of the region rather than the population density. Children and senior citizens were the most vulnerable to cold-related mortality, and these groups accounted for approximately 50.68% and 20.42% of cases, respectively. More men (256) died than women (174), and the primary cause of female deaths was burns from campfires. We believe that the total number of cold-related mortalities is higher than that reported in the hospital data used in this study, which is therefore considered to be a study limitation. Nevertheless, the results of this study will be helpful for understanding the importance of developing national policies and a framework for establishing special care and burn units in district hospitals to save vulnerable people from cold-related hazards in Bangladesh. However, we recommend a thorough evaluation of the methods used in this study before applying them to any policy-making process to mitigate cold-related mortality in Bangladesh.

## Figures and Tables

**Table 1:** Cold-related mortality occurring within 10-day periods in winter months in the divisions of Bangladesh during 2009–2021.

Period		Division	2009–2010	2010–2011	2011–2012	2012–2013	2013–2014	2014–2015	2015–2016	2016–2017	2017–2018	2018–2019	2019–2020	2020–2021	Total		
															Division	Period	Month
December	1–10	Chattogram									1				1	2	568
		Dhaka											1		1		
	11–20	Chattogram	9						1						10	57	
		Dhaka				4									4		
		Khulna			2			2							4		
		Rangpur			22	4									26		
		Sylhet										4		5	9		
		Across BD *			4										4		
	21–31	Barishal			1	1		21				110			133	509	
		Chattogram	3		3										6		
		Dhaka			8				1						9		
		Khulna	15		44	5	22	5							91		
		Mymensingh			15			2							17		
		Rajshahi			39	1		23							63		
		Rangpur			67	41	26		1		3		5	1	144		
		Sylhet			2	1							5		8		
		Across BD *											38		38		
January	1–10	Barishal											1		1	174	669
		Chattogram	5							1	3				9		
		Dhaka	5			13		3							21		
		Khulna	8	1		5					1				15		
		Mymensingh				1									1		
		Rajshahi	4		1	3		1			6				15		
		Rangpur	10	15		25	15	25			9	2			101		
		Sylhet				5					6				11		
	11–20	Barishal					3					7			10	287	
		Chattogram	24	8		7							6		45		
		Dhaka		3		5	2		3	3					16		
		Khulna	7	9				1		4	5				26		
		Mymensingh				3									3		
		Rajshahi	4	7				7		2	1				21		
		Rangpur	19	28	6	7	14		2	3	21	6	6	19	131		
		Sylhet	1	1		1				5					8		
		Across BD *				20					7				27		
	21–31	Barishal						21	6						27	208	
		Dhaka									4				4		
		Khulna						2	6		2				10		
		Rajshahi				19			88						107		
		Rangpur				12	2	3			7			27	51		
		Sylhet						4	5						9		
February	1–10	Khulna					1								1	12	12
		Mymensingh		3											3		
		Rangpur					2		1						3		
		Sylhet						5							5		
**Annual Total**			**114**	**75**	**214**	**183**	**87**	**125**	**114**	**18**	**76**	**129**	**62**	**52**	**1249**	**1249**	**1249**

* BD = Bangladesh.

**Table 2:** Causes of death during the winter months of 2009–2021.

Cause of Death		2009–2010	2010–2011	2011–2012	2012–2013	2013–2014	2014–2015	2015–2016	2016–2017	2017–2018	2018–2019	2019–2020	2020–2021	Total	
Cold		107	70	208	145	41	113	51	13	19	127	6	43	943	943
Disease	Pneumonia	5	5	3	23	6	6	2		11				61	133
	Diarrhoea			2				3				30		35	
	Asthma	2				14	5			2		14		37	
Burn				1	3	1	1	2	5	37	2	12	9	73	73
Unknown					12	25		56		7				100	100
Annual Total		114	75	214	183	87	125	114	18	76	129	62	52	1249	1249

**Table 3:** Death and hospitalization due to campfire-related burns in the divisions of Bangladesh during the winter months of 2009–2021.

Division	Death								Hospitalization				
	Age (Year)				Gender			Age and Gender Not Mentioned	Age (Year)				Age and Gender Not Mentioned
	6 and below	7–14	15–64	65 and above	Male	Female	Children		6 and below	7–14	15–64	65 and above	
Barishal			1			1							
Chittagong			3		2	1							
Dhaka			2	2	1	3		4					
Khulna	2		1	1	1	1	2						
Mymensingh								2					20
Rajshahi			1	3	1	3							
Rangpur	1		23	12	5	30	1	15	11		02		132
Total	3		31	18	10	39	3	21	11		02		152

## Data Availability

All the output data have been available in the manuscript in the form of figures and tables.

## Appendix A

**Table A1:** Gender distribution of cold-related deaths in the divisions of Bangladesh during the winter months of 2009–2021.

Gender	Division	2009–2010	2010–2011	2011–2012	2012–2013	2013–2014	2014–2015	2015–2016	2016–2017	2017–2018	2018–2019	2019–2020	2020–2021	Total	
														Division	Gender
Male	Barishal							4						4	256
	Chattogram	4			5			1	1	2				13	
	Dhaka	4	1	3	12	1			3					24	
	Khulna	18	5	18	5	1	6		3	2				58	
	Mymensingh			6	1		2							9	
	Rajshahi	5	5	16	3		6		1	3				39	
	Rangpur	6	11	50	13	4	1		2	4		3		94	
	Sylhet	1	1	1	5		3	1				3		15	
Female	Barishal											1		1	174
	Chattogram	2		2	2									6	
	Dhaka	1		3	2	1		4				1		12	
	Khulna	10	4	14	4	1	1	3	1	2				40	
	Mymensingh			5	0									5	
	Rajshahi	2	2	16	0		2		1					23	
	Rangpur	3	6	19	8	8	2	1	1	25	2	4	3	82	
	Sylhet				2		1					2		5	
Children	Barishal			1		3	39	2			117			162	633
	Chattogram	35		1						2		6		44	
	Dhaka			2	7									9	
	Khulna		1	13		21	3	1		1				40	
	Mymensingh		3	4										7	
	Rajshahi			3	20		23	65		4				115	
	Rangpur	3	24	25	45	38	17	3		7	6	1	38	207	
	Sylhet			1			5	4	5	6	4		5	30	
	Across BD *											19		19	
Gender not mentioned	Barishal				1		3							4	186
	Chattogram		8											8	
	Dhaka		2		1		3			4				10	
	Khulna	2		1	1			2		3				9	
	Mymensingh				3									3	
	Rajshahi	1		5				23						29	
	Rangpur	17	2	1	23	9	8			4		3	6	73	
	Across BD *			4	20					7		19		50	
Annual Total		114	75	214	183	87	125	114	18	76	129	62	52	1249	1249

* BD = Bangladesh.

**Table A2:** Age distribution of cold-related deaths in the divisions of Bangladesh during the winter months of 2009–2021.

Age (Year)	Division	2009–2010	2010–2011	2011–2012	2012–2013	2013–2014	2014–2015	2015–2016	2016–2017	2017–2018	2018–2019	2019–2020	2020–2021	Total	
														Division	Age Group
6 and below	Barisal			1		3	39	2			117			162	633
	Chattogram	35		1						2		6		44	
	Dhaka			2	7									9	
	Khulna		1	13		21	3	1		1				40	
	Mymensingh		3	4										7	
	Rajshahi			3	20		23	65		4				115	
	Rangpur	3	24	25	45	38	17	3		7	6	1	38	207	
	Sylhet			1			5	4	5	6	4		5	30	
	Across BD *											19		19	
7–14	Chattogram	1												1	7
	Khulna			1										1	
	Rajshahi			1			1							2	
	Rangpur			2	1									3	
15–64	Barisal											1		1	166
	Chattogram	1		1	3			1	1					7	
	Dhaka	2		4	8	2		3	2					21	
	Khulna	6	4	11	2	1	2		1	1				28	
	Mymensingh			4			2							6	
	Rajshahi	5	3	8						2				18	
	Rangpur	2	5	20	7	7	2			21	2	3	3	72	
	Sylhet			1	3		4	1				4		13	
65 and above	Barisal							4						4	255
	Chattogram	4		1	4					2				11	
	Dhaka	3	2	2	5			1	1			1		15	
	Khulna	24	5	19	6	1	5	3	3	3				69	
	Mymensingh			4	1									5	
	Rajshahi	2	4	24	3		7		2	1				43	
	Rangpur	7	12	46	14	5	1	1	3	8		4		101	
	Sylhet	1	1		4							1		7	
Age not mentioned	Barisal				1		3							4	188
	Chattogram		8											8	
	Dhaka		1		2		3			4				10	
	Khulna			2	2			2		3				9	
	Mymensingh			3	3									6	
	Rajshahi	1		4				23						28	
	Rangpur	17	2	2	22	9	8			4		3	6	73	
	Across BD *			4	20					7		19		50	
Annual Total		114	75	214	183	87	125	114	18	76	129	62	52	1249	1249

* BD = Bangladesh.
